# ESTs Analysis of Putative Genes Engaged in *Polyporus umbellatus* Sclerotial Development

**DOI:** 10.3390/ijms150915951

**Published:** 2014-09-10

**Authors:** Chao Song, Mengmeng Liu, Yongmei Xing, Shunxing Guo

**Affiliations:** Institute of Medicinal Plant Development, Chinese Academy of Medical Sciences and Peking Union Medical College, Beijing 100193, China; E-Mails: songchao0423@163.com (C.S.); liuermengmeng@hotmail.com (M.L.); meimary@163.com (Y.X.)

**Keywords:** *Polyporus umbellatus*, sclerotia, suppression subtractive hybridization (SSH) cDNA library, sequence analysis, real-time qPCR assay

## Abstract

*Polyporus umbellatus* is one of the most widely used and precious medicinal fungi and the underground sclerotia are known to be with great medicinal value. However, the molecular mechanisms involved in sclerotial development are poorly understood. In the present study, we constructed a forward suppression subtractive hybridization (SSH) cDNA library of *Polyporus umbellatus* to identify genes expressing differently between mycelium and sclerotia. In this library, a total of 1202 clones were sequenced, assembled into 222 contigs and 524 singletons which were further searched against the NCBI nonredundant (NR) protein database (*E*-value cutoff, 10^−5^). Based on sequence similarity with known proteins, 378 sequences between mycelium and sclerotial were identified and classified into different functional categories through Gene Ontology (GO), Clusters of orthologous Groups of proteins (COGs). We have finally identified a majority of differentially expressed genes (constituting 5.6% of the present library) between the two different periods. An expression level of 32 selected expressed sequence tags (ESTs) generated from the above SSH cDNA library was studied through RT-PCR. This study provides the first global overview of genes putatively involved in *Polyporus umbellatus* sclerotial development and provides a preliminary basis for further functional research in terms of regulated gene expression in sclerotial production.

## 1. Introduction

*Polyporus umbellatus*, also known as *Grifola umbellata*, belongs to Polyporaceae in the class of Basidiomycetes and as a species of wood-rotting fungi [1]. *P. umbellatus* forms sclerotia underground, and it distributes in broad-leaved forests in Japan, China, Korea, and temperate regions of the Northern Hemisphere [2]. *P. umbellatus* can survive for long periods of time below soil, and produce new sclerotia directly from the old ones under appropriate conditions [3]. *P. umbellatus* has been used as a natural medicine in China for more than 2500 years. It has been demonstrated that *P. umbellatus* has significant pharmacological effects in treating edema, acute nephritis and diarrhea [4]. In addition, *P. umbellatus* has been verified with various pharmacological activities such as anti-tumor activity, diuretic activity and treatment of chronic kidney disease [5,6,7,8,9,10,11,12,13]. The compounds that isolated from *P. umbellatus* such as ergosterol, d-mannitol are both with diuretic effects, Polyporusterone A, B, C, D, E, F, G are all with anticancer activity [14,15,16]. However, limited natural resources, over-harvesting and severe habitat loss threaten the survival of the species. Therefore, it is urgent to promote mass production of sclerotia under artificial conditions. Although semi-artificial cultivation of *P. umbellatus* via infection with *Armillaria mellea* has become mature over the past 30 years, the low proliferation rate and unstable yield limit the speed of its promotion [17]. Thus, much interest has been focused on *P. umbellatus* sclerotial produced directly from hyphae instead of old sclerotia in the laboratory conditions.

In our previous work, we successfully induced sclerotial production from mycelium on artificial media, which contains maltose, fructose and glucose, and at the optimum pH 5.0 [4]. Furthermore, we have demonstrated that the split-plate culture method was an effective way to induce *P. umbellatus* sclerotial production [18] and Ca^2+^ signal transduction was found to play an important role in *P. umbellatus* sclerotial formation [19]. These attempts have opened up the possibility to understand the explicit factors affecting *P. umbellatus* sclerotial development. Even though much work have been carried out for the induction of sclerotium from hyphae of *P. umbellatus* under artificial conditions, analysis of the molecular mechanisms involved in sclerotial development is still in its infancy. Thus, identifying the molecular factors affecting sclerotial development and determining their roles will be beneficial for protecting the wild *P. umbellatus* sclerotial resources. In turn, this will enable us to control the development of *P. umbellatus* on the one hand, and improve the pharmaceutical exploitation of *P. umbellatus* sclerotia, on the other. To elucidate the molecular mechanism of sclerotial development of *P. umbellatus*, we constructed a suppression subtractive hybridization (SSH) cDNA library enriched for genes specifically expressed between the mycelium and sclerotia. Differentially expressed genes were examined by sequencing, analyzing expressed sequence tags (ESTs) present in the library and quantitative real-time polymerase chain reaction (qRT-PCR) analysis. These new EST resources are an important addition to publically available resource especially in relation to the study of sclerotial development.

## 2. Results and Discussion

### 2.1. Morphological Identification

**Figure 1:**
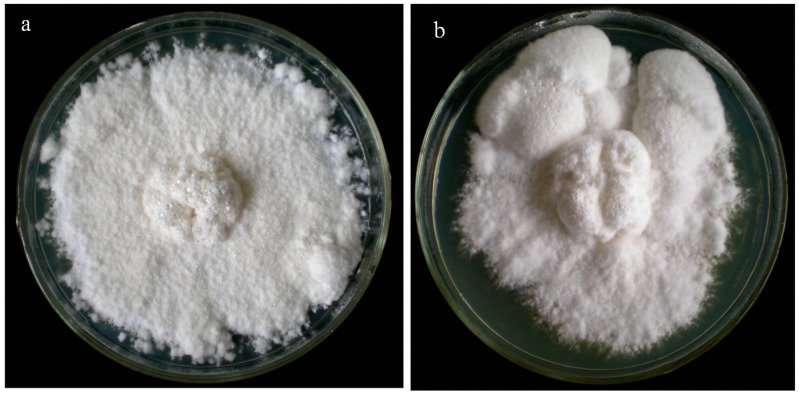
Inducing sclerotial formation under artificial conditions. (**a**) Mycelia could not form sclerotia; (**b**) sclerotial formation after 60 days cultivation.

After cultivation for 30 days, as shown in Figure 1a, the hyphe were almost full of the 9-cm plate without forming sclerotial structures. Snowwhite interwoven hyphae with abundant aerial mycelia appeared at day 60 of cultivation (Figure 1b) which indicated that the *P. umbellatus* sclerotial were successful induced in the artificial conditions.

### 2.2. EST (Expressed Sequence Tag) Assembly

From the forward-subtracted library, a total of 1202 EST sequences were obtained and analyzed. A total of 678 ESTs (56% of the total number of ESTs) were assembled into 222 contigs, each composed of 2–41 sequences. The contigs for the remaining 524 ESTs contained only single ESTs (singletons). Comparison of the sequences against the BLASTX and only with *E*-values smaller than 10^−5^ were retained as significant. The result showed that 378 contigs (51% of the total ESTs) were functionally annotated where 192 contigs (26% of the total ESTs) annotated with hypothetical genes or genes with unknown functions and 176 uniseqs were with no hits or with poor similarity (*E*-values ≥ 10^−5^). Most of the ESTs showed high similarity to basidiomycete and just a few showed similarity to ascomycete and bacteria. All the sequences have been uploaded to the NCBI database (NIH, Bethesda, MD, USA), EST subdatabase.

### 2.3. Functional Characterization of EST Data Set

**Figure 2:**
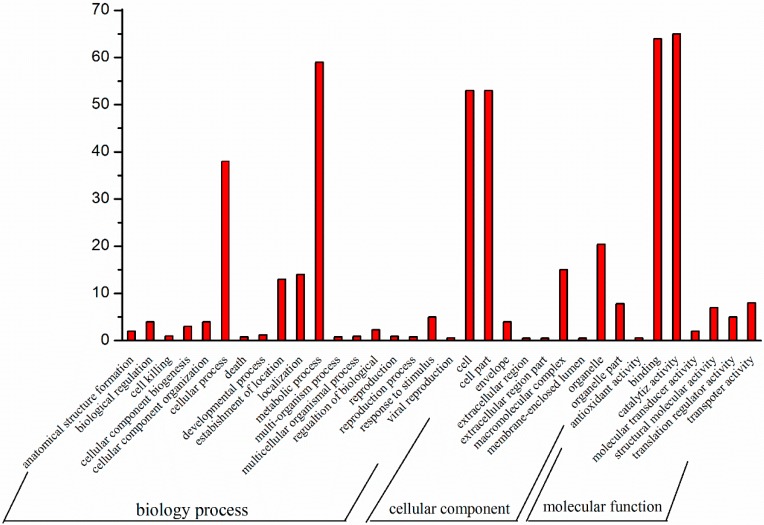
Representation of gene ontology assignments for fungal homologous expressed sequence tags (ESTs) derived from the suppression subtractive hybridization (SSH) library.

Based on GO annotation, gene products can be assigned to three organizing principal GO categories: molecular functions, cellular components and biological processes [20]. As shown in Figure 2, in biological processes, the metabolic process was the dominant part, followed by cellular process and localization. In the molecular function category, catalytic activity was the most dominant term, followed by binding function and in the cellular components, cell part was the most represented term.

**Figure 3:**
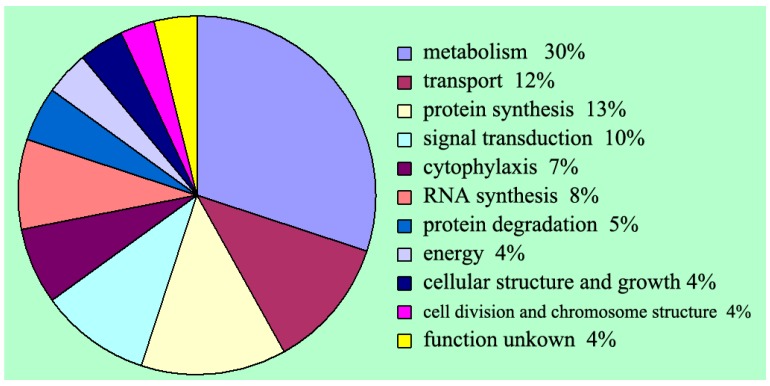
A graphical representation of sequence distribution based on Clusters of orthologous Groups of proteins (COGs).

The database of Clusters of Orthologous Groups of proteins (COGs) is a phylogenetic classification of proteins encoded in completely sequenced genomes [21]. As the COG system is designed for functional annotation of genomes, a tagged protein can be clustered into COGs by comparing with all proteins in the COGs. We mapped the COGs database for fungal homologous proteins to group the sequenced uniseqs with known function based on the SWISS-PROT database (University of Geneva, Geneva, Switzerland). It was shown that 378 uniseqs displayed significant hit with COGs database and grouped into 11 functional categories (Figure 2). As shown in Figure 3, the metabolic process was the dominant part (30%), followed by protein biosynthesis (13%) and transport (12%), which indicated the positively vital activity during the process of *P. umbellatus* sclerotial development.

### 2.4. Transcription Expression Patterns of Candidate Genes through RT-PCR Assay

**Figure 4:**
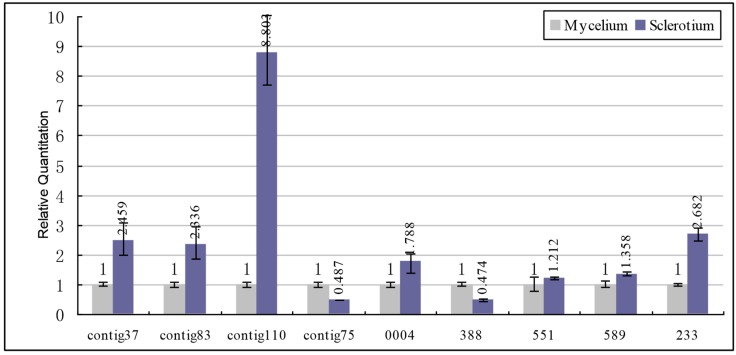
Expression patterns of redox related genes from SSH library using qPCR analyses. Blue columns represent the Relative Quantification (RQ) in sclerotium; light grey columns indicate the RQ in mycelium. All standards were run in duplicate and samples were run in triplicate. Contig37: glyoxal oxidase; Contig83: choline oxidase; Contig110: NADPH oxidase; Contig75: glutathione peroxidase; 0004: peroxisomal membrane protein; 388: cysteine peroxidase; 551: thioredoxin-dependent peroxidase; 589: chloroperoxidase-like protein; 233: alcohol oxidase-like protein.

We verify the expression patterns of 32 differentially expressed genes based on their putative functions and the results of the SSH library using real-time qPCR. These genes are involved in oxidative stress which can induce sclerotial metamorphosis in filamentous fungi [22], such as contig37 (glyoxal oxidase), Contig83 (choline oxidase), 233 (alcohol oxidase-like protein), (NADPH oxidase isoform 1), Contig75 (glutathione peroxidase), 0004 (peroxisomal membrane protein), 388 (cysteine peroxidase), 551 (thioredoxin-dependent peroxidase), 589 (chloroperoxidase-like protein). As shown in Figure 4, all selected genes which can encode into oxidase-like proteins show a significant upregulation in sclerotium except for the peroxidase-like genes (Contig75, Pu388, Pu551, Pu589 and Pu0004). It has also been demonstrated that reactive oxygen species (ROS) plays a key role in multi-cellular differentiation, fruiting body development and ascospore germination in fungi [23]. NADPH oxidase (Contig110) and glyoxal oxidase (Contig37) have been considered as candidates for ROS generation [24,25]. Upregulation of these two genes indicate the significant ROS production in sclerotium. Meanwhile, glutathione peroxidase (Contig75) and cysteine peroxidase (388) displaying extremely high peroxide decompose and antioxidant activity respectively are downregulated in sclerotium [26]. Furthermore, it has been shown that the inhibitor of catalase activity inhibit the development of *Sclerotium rolfsii* sclerotium and sclerotial initials show100-fold increase in lipid peroxides of their total lipids as compared with young mycelia in *Sclerotium rolfsii* [13,27]. Therefore, it was assumed that the sclerotium adapts to oxidative stress by upregulating oxidation and downregulating antioxidant related gene expression.

**Figure 5:**
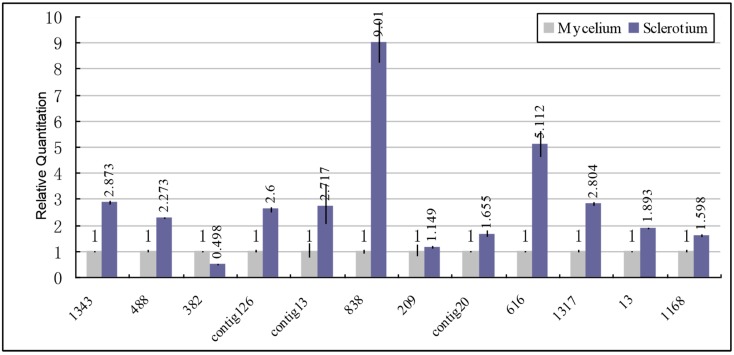
Expression patterns of signal related genes from SSH library using qPCR analyses. Blue columns represent the RQ in sclerotium; light grey columns indicate the RQ in mycelium. All standards are run in duplicate and samples are run in triplicate. Puctg838, Puctg616, Puctg1343, Pu13, Pu1168: small GTPase; Puctg20, Puctg13, Pu1317, Pu382, Pu488: protein kinase; Puctg126: G protein; Pu209: G protein-coupled receptor (GPCR).

As shown in the Figure 5, it is interesting to find that in our study the signal transduction related genes show a significant upregulation in sclerotium such as G protein (2.6-fold), small GTPase (Puctg838, Puctg616, Puctg1343, Pu13, Pu1168), Mitogen-activated protein kinase (MAPK) (2.804-fold), Kinase Group AGC (1.655-fold), Casein Kinases (2.273-fold) and protein kinase 1 (PK1) (2.717-fold). However, the expression of Glycogen synthase kinase (GSK) protein kinase is downregulated in sclerotium. It has been strongly suggested that MAPK activaty and protein kinase A (PKA) activity is involved in sclerotial development [28,29]. Sclerotium experienced increased expression of MAPK, AGC, Casein Kinases and PK1 as compared with mycelium which suggests that the possibility of the involvement in sclerotium development. Moreover, among the above genes, the small GTPase specifically involves in gene expression, cytoskeleton formation, differentiation, transport and location [30], which shows a significant upregulation in sclerotium. Therefore, it is reasonable to assume that the upregulating of small GTPase may be involved in sclerotial development through different signaling pathways.

**Figure 6:**
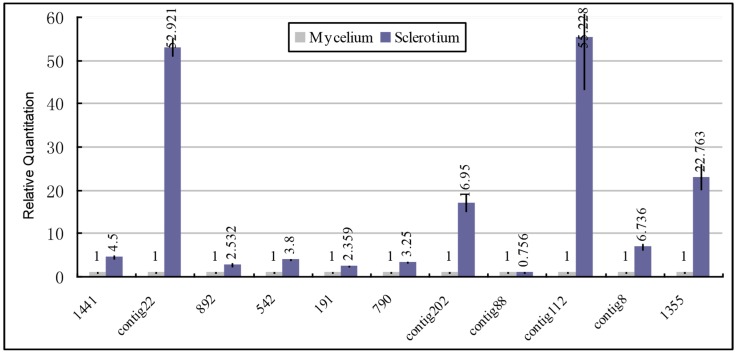
Expression patterns of growth related genes from SSH library using qPCR analysis. Blue columns represent the RQ in sclerotium; light grey columns indicate the RQ in mycelium. All standards were run in duplicate and samples were run in triplicate. Pu1441: cytochrome P450; Puctg22, Puctg8: hemolysin; Pu892, Pu542: inhibitor of apoptosis; Pu191: Cyclins, Pu790: cyclophilin, Puctg202, Puctg112: hydrophobin; Pu1355: C-4 sterol methyl oxidase; Puctg88: hydrophobin.

It has been reported that many genes, such as cytochrome P450, C-4 sterol methyl oxidase, hydrophobin and hemolysin specifically express in the fruit body of advanced fungus [31,32,33]. In our project, we find that the genes related to development and growth of the advanced fungus such as encoding cytochrome P450 (Pu1441), hemolysin (Puctg22, Puctg8), inhibitor of apoptosis (Pu892, Pu542), cyclins (Pu191), cyclophilin (Pu790), hydrophobin (Puctg202, Puctg112) and C-4 sterol methyl oxidase (Pu1355) (Figure 6) all maintained a higher level of expression in sclerotia except the hydrophobin (Puctg88). The possibility exists in that these differentially upregulated genes contribute to the sclerotium development. However, to substantiate this hypothesis, a detailed exploration of their role in the sclerotium development needs to be conducted in the future.

## 3. Experimental Section

### 3.1. Sclerotial Material Preparation

In this study, *P. umbellatus* was incubated in fructose complete medium [4] at 25 °C under dark conditions for sclerotial formation. After 60 days, sclerotium started to develop. Mycelia samples (defined as H) and sclerotia samples (defined as S) were collected in one plate. All the samples were immediately frozen in liquid nitrogen and stored at −80°C prior to total RNA extraction.

### 3.2. RNA and mRNA Isolation

Total RNA was isolated using the CTAB (Cetyl Trimethyl Ammonium Bromide)-LiCl protocol with some modification that we added 10 mL CTAB-LiCl buffer per 0.5 g of fresh weight tissue as the materials contained much polysaccharide [34], and messenger RNA (mRNA) was further isolated by using the PolyATract mRNA Isolation system (Promega, Madison, WI, USA) [35]. Quantity and quality of mRNA was estimated using NanoDrop 2000c (Thermo Scientific, Wilmington, DE, USA).

### 3.3. Suppression Subtractive Hybridization (SSH)

SSH cDNA library [36] was constructed by using the Clontech PCR-Select™ cDNA subtraction kit (Clontech, Palo Alto, CA, USA) following manufacturer’s instructions, using 2 μg of mRNA from tester and driver samples. The differential PCR products generated by SSH were inserted in the pGEM-T Vector (Promega, Madison, WI, USA) and the constructs were transformed into competent DH5α *E. coli* (Trans, Beijing, China).

### 3.4. cDNA Sequencing and EST Analyses

All the insert-positive clones from the two libraries were sequenced by Beijing Genewiz, Inc. (Beijing, China) using the Sanger sequencing platform. The vector and adaptor fragments were removed by SecMan II sequence analysis software (DNAStar Inc., Madison, WI, USA). The obtained sequence data comprised consensus sequences and singletons were manually scrutinized for open reading frames (ORFs) using the NCBI ORF-finder [37]. All the inserted sequences were checked for homologies in the GenBank database using BLASTX sequence comparison software at the website [38]. Significant similarity scores of *E*-value of 1 × 10^−5^ (a default vlaue) were counted as a hit. Contigs were grouped into functional categories based on the Gene Ontology database [39], and gene expression was analyzed via EST sampling using COG database [40].

### 3.5. Real-Time qPCR Analysis

To verify the quality of the SSH library and analyze the characterization of gene expression in mycelium and sclerotia, target mRNAs, identified using SSH, were selected for further investigation using real-time quantitative RT-PCR. The real-time PCR was performed with the SYBR^®^ Premix Ex TaqTM (TaKaRa, Dalian, China) on the ABI 7500 Real-Time PCR System (Applied Biosystems, Foster City, CA, USA). Generated cDNAs were diluted at 1/10 with sterile water performing the qPCR assay. Real-time PCR was carried out in triplicates in a total volume of 25 μL containing 12.5 μL 2× SYBR^®^ Premix Ex TaqTM Master Mix, 0.5 μL each primer (10 μM), 0.5 μL ROX Reference Dye, 2 μL cDNA template, and 9 μL double distilled water. The thermal conditions were as follows: 95 °C for 30 s, followed by 40 cycles of 95 °C 5 s, 60 °C 34 s. The ribosomal 18S was chosen to evaluate stability as endogenous control gene using the BestKeeper method described by Pfaffl *et al.* [41]. A melting curve of PCR products were performed to ensure the detection of a single specific product. Cycle threshold (*C*_t_) values were generated from the ABI PRISM 7500 Software Tool (Applied Biosystems, Foster City, CA, USA). The relative transcript levels were calculated using the comparative ∆∆*C*_t_ method of relative gene quantification [42]. *U*-Values equal to 0 and *p* values lower than 0.05 were used to identify significant differences.

## 4. Conclusions

In the present project, we reported a combination of SSH library and real-time qPCR techniques to determine gene expression patterns during sclerotium production. To the best of our knowledge, this is the first molecular study of *Polyporus umbellatus* towards the genes putatively involved in *P. umbellatus* sclerotial development. In the SSH library, a variety of fungal homologous ESTs are classified and most are assigned as sclerotium production related. These Unigenes are identified, annotated and found to participate in many biological processes, such as metabolic process, protein biosynthesis and transport. In conclusion, thirty-two specific genes are identified between the morphologically distinct mycelium and sclerotial.

In addition, the RT-PCR analysis showed that the differentially express genes of oxidative stress related genes with known function and MAPK activity maintain a higher level of expression in the sclerotial period of *P. umbellatus*. These distinctive and differentially expressed genes may play important roles in development, growth, metabolism, and proliferation. Further characterization of these distinct genes will provide useful information to further elucidate the molecular events regulating sclerotia production.

## References

[1] Zhao Y.-Y. (2013). Traditional uses, phytochemistry, pharmacology, pharmacokinetics and quality control of *Polyporus umbellatus* (Pers.) Fries: A review. J. Ethnopharmacol.

[2] Lee J. (1988). Colored Korean Mushrooms.

[3] Xu J. (1997). Chinese Medicinal Mycology.

[4] Xing Y.-M., Chen J., Lv Y.-L., Liang H.-Q., Guo S.-X. (2011). Determination of optimal carbon source and pH value for sclerotial formation of *Polyporus umbellatus* under artificial conditions. Mycol. Prog..

[5] Sun Y., Ji Z., Zhao Y., Liang X., Hu X., Fan J. (2013). Enhanced distribution and anti-tumor activity of ergosta-4,6,8(14),22-tetraen-3-one by polyethylene glycol liposomalization. J. Nanosci. Nanotechnol..

[6] Yuan D., Mori J., Komatsu K.-I., Makino T., Kano Y. (2004). An anti-aldosteronic diuretic component (drain dampness) in *Polyporus sclerotium*. Biol. Pharm. Bull..

[7] Zhao Y.-Y., Xie R.-M., Chao X., Zhang Y., Lin R.-C., Sun W.-J. (2009). Bioactivity-directed isolation, identification of diuretic compounds from *Polyporus umbellatus*. J. Ethnopharmacol..

[8] Zhao Y.-Y., Shen X., Chao X., Ho C.C., Cheng X.-L., Zhang Y., Lin R.-C., Du K.-J., Luo W.-J., Chen J.-Y. (2011). Ergosta-4,6,8(14),22-tetraen-3-one induces G2/M cell cycle arrest and apoptosis in human hepatocellular carcinoma HepG2 cells. Biochim. Biophys. Acta BBA Gen. Subj..

[9] Zhao Y.-Y., Zhang L., Mao J.R., Cheng X.H., Lin R.C., Zhang Y., Sun W.J. (2011). Ergosta-4,6,8(14),22-tetraen-3-one isolated from *Polyporus umbellatus* prevents early renal injury in aristolochic acid-induced nephropathy rats. J. Pharm. Pharmacol..

[10] Zhao Y.-Y., Cheng X.-L., Cui J.-H., Yan X.-R., Wei F., Bai X., Lin R.-C. (2012). Effect of ergosta-4,6,8(14),22-tetraen-3-one (ergone) on adenine-induced chronic renal failure rat: A serum metabonomic study based on ultra performance liquid chromatography/high-sensitivity mass spectrometry coupled with MassLynx i-FIT algorithm. Clin. Chim. Acta.

[11] Chen D.-Q., An J.-M., Feng Y.-L., Tian T., Qin X.-Y., Zhao Y.-Y. (2013). Cloud-point extraction combined with liquid chromatography for the determination of ergosterol, a natural product with diuretic activity, in rat plasma, urine, and feces. J. Anal. Methods Chem..

[12] Zhao Y.-Y., Zhang L., Long F.-Y., Cheng X.-L., Bai X., Wei F., Lin R.-C. (2013). UPLC-Q-TOF/HSMS/MS^E^-based metabonomics for adenine-induced changes in metabolic profiles of rat feces and intervention effects of ergosta-4,6,8(14),22-tetraen-3-one. Chem. Biol. Interact..

[13] Papapostolou I., Sideri M., Georgiou C.D. (2013). Cell proliferating and differentiating role of H_2_O_2_ in Sclerotium rolfsii and Sclerotinia sclerotiorum. Microbiol. Res..

[14] Zhao Y.-Y., Chao X., Zhang Y., Lin R.-C., Sun W.-J. (2010). Cytotoxic steroids from *Polyporus*. Planta Med..

[15] Zhao Y.-Y., Cheng X. L., Zhang Y., Zhao Y., Lin R.C., Sun W.J. (2010). Simultaneous determination of eight major steroids from *Polyporus umbellatus* by high-performance liquid chromatography coupled with mass spectrometry detections. Biomed. Chromatogr..

[16] Peng K., Lan L.S., Yan W.X., Jie S.L., Wu Y.J., Hua Z.Y., Shu-Chen L. (2012). *Polyporus umbellatus* polysaccharides ameliorates carbon tetrachloride-induced hepatic injury in mice. Afr. J. Pharm. Pharmacol..

[17] Liu Y.-Y., Guo S. (2009). Nutritional factors determining sclerotial formation of *Polyporus umbellatus*. Lett. Appl. Microbiol..

[18] Huang H.-C., Liu Y.-C. (2008). Enhancement of polysaccharide production by optimization of culture conditions in shake flask submerged cultivation of *Grifola umbellata*. J. Chin. Inst. Chem. Eng..

[19] Liu Y.-Y., Guo S.-X. (2010). Involvement of Ca^2+^ channel signaling in sclerotial formation of *Polyporus umbellatus*. Mycopathologia.

[20] Berardini T.Z., Mundodi S., Reiser L., Huala E., Garcia-Hernandez M., Zhang P., Mueller L.A., Yoon J., Doyle A., Lander G. (2004). Functional annotation of the Arabidopsis genome using controlled vocabularies. Plant Physiol..

[21] Tatusov R.L., Koonin E.V., Lipman D.J. (1997). A genomic perspective on protein families. Science.

[22] Scott B., Eaton C.J. (2008). Role of reactive oxygen species in fungal cellular differentiations. Curr. Opin. Microbiol..

[23] Takano M., Nakamura M., Yamaguchi M. (2010). Glyoxal oxidase supplies hydrogen peroxide at hyphal tips and on hyphal wall to manganese peroxidase of white-rot fungus Phanerochaete crassa WD1694. J. Wood Sci..

[24] Niimura Y. (2007). The NADH oxidase-prx system in *Amphibacillus Xylanus*. Peroxiredoxin Systems.

[25] Harel A., Gorovits R., Yarden O. (2005). Changes in protein kinase A activity accompany sclerotial development in Sclerotinia sclerotiorum. Phytopathology.

[26] Dupuy C., Virion A., Ohayon R., Kaniewski J., Deme D., Pommier J. (1991). Mechanism of hydrogen peroxide formation catalyzed by NADPH oxidase in thyroid plasma membrane. J. Biol. Chem..

[27] Georgiou C.D. (1997). Lipid peroxidation in *Sclerotium rolfsii*: A new look into the mechanism of sclerotial biogenesis in fungi. Mycol. Res..

[28] Chen C., Dickman M.B. (2005). cAMP blocks MAPK activation and sclerotial development via Rap-1 in a PKA-independent manner in *Sclerotinia sclerotiorum*. Mol. Microbiol..

[29] Bucci C., Parton R.G., Mather I.H., Stunnenberg H., Simons K., Hoflack B., Zerial M. (1992). The small GTPase rab5 functions as a regulatory factor in the early endocytic pathway. Cell.

[30] Kennedy M.A., Johnson T.A., Lees N.D., Barbuch R., Eckstein J.A., Bard M. (2000). Cloning and sequencing of the *Candida albicans* C-4 sterol methyl oxidase gene (*ERG25*) and expression of an *ERG25* conditional lethal mutation in *Saccharomyces cerevisiae*. Lipids.

[31] Hirano T., Sato T., Enei H. (2004). Isolation of genes specifically expressed in the fruit body of the edible basidiomycete *Lentinula edodes*. Biosci. Biotechnol. Biochem..

[32] Sunagawa M., Magae Y. (2005). Isolation of genes differentially expressed during the fruit body development of *Pleurotus ostreatus* by differential display of RAPD. FEMS Microbiol. Lett..

[33] De Groot P.W., Schaap P.J., van Griensven L.J., Visser J. (1997). Isolation of developmentally regulated genes from the edible mushroom *Agaricus bisporus*. Microbiology.

[34] Iandolino A., da Silva F.G., Lim H., Choi H., Williams L., Cook D. (2004). High-quality RNA, cDNA, and derived EST libraries from grapevine (*Vitis vinifera* L.). Plant Mol. Biol. Report..

[35] Deokar A.A., Kondawar V., Jain P.K., Karuppayil S.M., Raju N., Vadez V., Varshney R.K., Srinivasan R. (2011). Comparative analysis of expressed sequence tags (ESTs) between drought-tolerant and-susceptible genotypes of chickpea under terminal drought stress. BMC Plant Biol..

[36] Houde M., Belcaid M., Ouellet F., Danyluk J., Monroy A.F., Dryanova A., Gulick P., Bergeron A., Laroche A., Links M.G.  (2006). Wheat EST resources for functional genomics of abiotic stress. BMC Genomics.

[37] ORF Finder. http://www.ncbi.nlm.nih.gov/gorf/gorf.html.

[38] BLASTX. http://www.ncbi.nlm.nih.gov/BLAST.

[39] Gene Ontology Consortium. http://www.geneontology.org.

[40] COGs—Clusters of Orthologous Groups. http://www.ncbi.nlm.nih.gov/COG/.

[41] Pfaffl M.W., Tichopad A., Prgomet C., Neuvians T.P. (2004). Determination of stable housekeeping genes, differentially regulated target genes and sample integrity: BestKeeper-Excel-based tool using pair-wise correlations. Biotechnol. Lett..

[42] Pfaffl M.W. (2001). A new mathematical model for relative quantification in real-time RT–PCR. Nucleic Acids Res..

